# Conformational Analysis of 3-Indoleacetamide: Unveiling Structural Rigidity in the Tryptophan-Derived Bioactive Molecule Family

**DOI:** 10.3390/molecules30214156

**Published:** 2025-10-22

**Authors:** Sofía Municio, Sergio Mato, José Luis Alonso, Elena Rita Alonso, Iker León

**Affiliations:** Grupo de Espectrocopía Molecular (GEM), Edificio Quifima, Laboratorios de Espectroscopia y Bioespectroscopia, Unidad Asociada CSIC, Parque Científico UVa, Universidad de Valladolid, 47011 Valladolid, Spain; sofia.municio@estudiantes.uva.es (S.M.); sergio.mato@uva.es (S.M.); jlalonso@uva.es (J.L.A.); elenarita.alonso@uva.es (E.R.A.)

**Keywords:** 3-indoleacetamide, plant hormone, Fourier transform microwave spectroscopy, structure-property, tryptophan derivatives

## Abstract

The conformational landscape of 3-indoleacetamide, a key intermediate in plant hormone biosynthesis, has been comprehensively investigated using state-of-the-art laser-ablation chirped-pulse Fourier transform microwave (LA-CP-FTMW) and laser-ablation molecular beam Fourier transform microwave (LA-MB-FTMW) spectroscopy. Remarkably, 3-indoleacetamide exhibits unprecedented conformational rigidity within the tryptophan-derived molecule family, displaying only a single stable conformer characterized by distinctive *a*-, *b*-, and *c*-type rotational transitions. This singular conformational behavior contrasts dramatically with the structural flexibility observed in closely related tryptophan derivatives such as tryptophan, serotonin, tryptamine, and 3-indoleacetic acid. The unique structural constraint imposed by the acetamide functional group provides unprecedented insights into the molecular determinants governing the distinct biological roles of tryptophan-derived compounds. This work establishes a potential correlation between conformational flexibility and biological function, from neurotransmission to plant hormone regulation, offering new perspectives on structure-activity relationships in bioactive natural products.

## 1. Introduction

**Figure 1:**
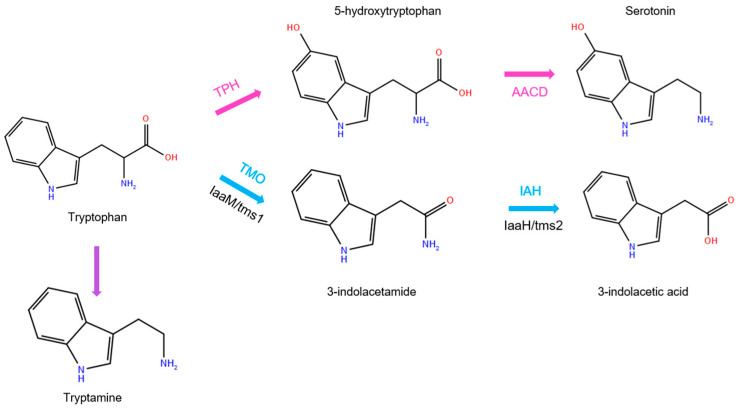
Two-step tryptophan-dependent serotonin (**top**) and auxin (**bottom**) biosynthesis pathways.

Tryptophan stands as one of nature’s most versatile amino acid precursors, serving as the biosynthetic foundation for an extraordinary array of bioactive molecules with diverse and specialized physiological functions [1,2,3]. This aromatic amino acid undergoes complex metabolic transformations that give rise to compounds ranging from essential neurotransmitters to critical plant growth regulators. Among the most significant metabolic pathways, as shown in Figure 1, are those leading to serotonin (5-hydroxytryptamine) [4,5], a pivotal neurotransmitter in the central nervous system, and 3-indoleacetic acid (IAA) [5,6,7], the primary auxin responsible for plant growth and development [8,9].

As shown in Figure 1, one of the biosynthetic pathways from tryptophan to IAA involves 3-indoleacetamide (IAM) serving as a crucial metabolic intermediate in one of the major routes [10]. This pathway represents a fascinating example of how nature has evolved distinct molecular architectures to achieve specific biological outcomes, despite starting from a common precursor.

The relationship between molecular structure and biological function has emerged as a fundamental principle in understanding biochemical processes and drug design. In the context of tryptophan-derived molecules, this relationship becomes particularly intriguing due to the subtle yet profound structural modifications that lead to dramatically different biological activities. Understanding the conformational preferences of these molecules is important because this is due to their intramolecular interactions and potential intermolecular interactions which result in their ability to adopt specific three-dimensional arrangements, thus playing a crucial role in determining their interactions with biological targets.

Gas phase experiments provide a unique and ideal medium to study, understand and model the interactions between small molecules free from any external interaction [11,12,13,14]. Microwave spectroscopy is one of the most powerful techniques that allows an unambiguous identification of the molecular structure, and hence the structural arrangement of the system under study [15,16,17]. Therefore, combining gas phase experiment with microwave spectroscopy offers a powerful approach to study the conformational panorama of biomolecules and their main intramolecular interactions.

Using this strategy previous spectroscopic investigations have established distinct conformational profiles for key members of the tryptophan family. Tryptamine displays remarkable conformational diversity as it adopts four conformational states [18]. This multiplicity arises from the inherent flexibility of the ethylamine chain and its differential interactions with both the benzene and pyrrole rings of the indole system. Serotonin is structurally similar to triptamine but with a phenolic hydroxyl group and also adopts four conformational states [19,20]. In this case, the phenolic hydroxyl group introduces additional conformational possibilities due to its orientation. Tryptophan on the other hand exhibits two stable conformers, both featuring the carboxyl group in trans configuration [21,22,23,24]. The inclusion of the carboxyl group with respect to tryptamine and serotonin reduces the flexibility of the ethylamine chain. In both conformers there is a hydrogen bond between the OH group and the amino group (O-H•••N), with one of the conformers stabilized by an intramolecular interaction between the amino group and the π-electron system of the pyrrole ring, and the second conformer involving reorientation of the amino group toward the phenolic ring.

Regarding 3-Indoleacetic acid, it presents a complex conformational landscape with four observed conformers (just submitted). Three of these maintain the carboxyl group in cis configuration but differ in their spatial orientation, with one conformer being planar and two out-of-plane. The conformational diversity stems from the ability of either carbonyl oxygen or hydroxyl oxygen to serve as hydrogen bond acceptors from aromatic C-H donors. A fourth, higher-energy conformer features the trans-carboxyl configuration with the hydroxyl group interacting with the π-electron density of the five-membered pyrrole ring.

In this context, we have performed the first rotational spectroscopic study of 3-Indoleacetamide using LA-CP-FTMW and LA-MB-FTMW spectroscopies to identify the most relevant conformers of this crucial metabolic intermediate. These precise structural results will be compared with those of tryptophan, tryptamine, serotonin and IAA.

## 2. Results and Discussion

### 2.1. Conformational Space

**Figure 2:**
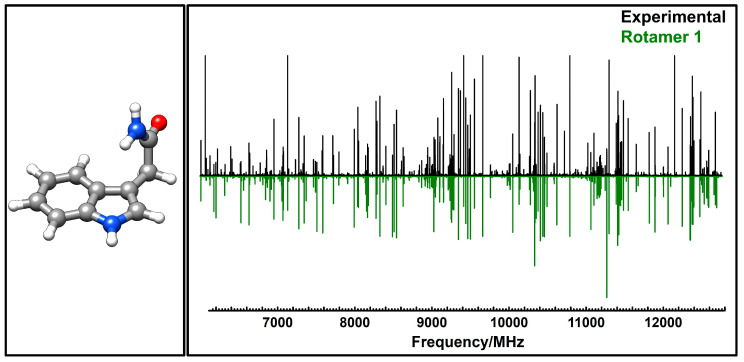
Left: Structure of the single conformer of IAM. Right: broadband experimental rotational spectrum of IAM in the 6000 to 13,000 MHz range (black), together with the calculated rotational transitions (green).

To identify the most energetically favorable geometries of neutral IAM, we performed an extensive conformational analysis using molecular mechanics approaches. This search yielded 12 distinct structures spanning an energy window of 2500 cm^−1^ (30 kJ/mol). Each structure underwent geometry optimization via quantum mechanical methods implemented in the Gaussian program suite [25], following the computational protocol detailed in the methodology. All 12 initial conformers ultimately relaxed to a single, lowest-energy structure. Figure 2 displays this optimized conformer, with the corresponding calculated spectroscopic constants at various theoretical levels presented in Table 1. As can be seen in Figure 2, the structure of the single conformer is stabilized by resonance delocalization within the acetamide group and a N–H•••π interaction between the amino NH_2_ group and the high π density sites of the pyrrolic ring.

### 2.2. Rotational Spectrum and Conformational Identification

Figure 2 presents the broadband microwave spectrum [26,27] of IAM recorded across the 6–13 GHz frequency range. After eliminating spectral contributions from known photodissociation fragments, we analyzed the spectrum using the predicted parameters for our single conformer. The substantial *µₐ* electric dipole moment facilitated identification of distinctive group of lines of *a*-type *R*-branch transitions, appearing at regular *B* + *C* intervals throughout the broadband survey. Initial fitting [28,29,30,31,32] of these transitions to a rigid rotor model provided refined rotational constants, enabling more accurate frequency predictions. This iterative approach allowed assignment of additional *a*-type transitions alongside *b*- and *c*-type transitions. Final analysis employed Watson’s asymmetric top semi-rigid rotor Hamiltonian using *A*-reduction in the Iʳ-representation [32]. The complete list of assigned transitions appears in Table S2, with the derived rotational parameters summarized in Table 1. The individual lines appeared broadened by the effect of the ^14^N nuclear quadrupole coupling interaction. For *µ_b_*- and *µ_c_*-type transitions the resolution of the instrument was enough to resolve some hyperfine components and make an initial determination of the quadrupole coupling constants. Nevertheless, because the values of the quadrupole coupling constants will be important for discussion, we also obtained the rotational spectra of selected lines using the LA-MB-FTMW spectrometer.

Thus, in a second step, we took advantage of the sub-Doppler resolution of the cavity-based LA-MB-FTMW technique [33,34] to resolve the hyperfine structure of the obtained rotamer. A total of 56 hyperfine components were measured (see Table S3). The spectroscopic parameters obtained are collected in Table 1.

### 2.3. The Acetamide N-H•••π Interaction

**Figure 3:**
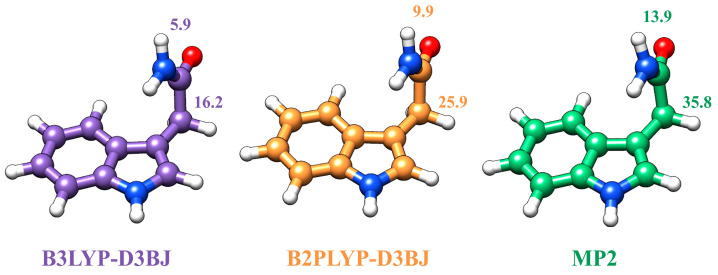
Comparison between the structure of the single conformer of IAM at different methodologies, all with the 6-311++G(d,p) basis set. The values indicate the O=C-N-H (**top**) and C-C-C-N (**right**) dihedral angles.

The comparison between the experimental and calculated parameters in Table 1 shows that when using a standard 6-311++G(d,p) Pople basis set, B3LYP-D3BJ calculations yield the most accurate results, with rotational constants *A*, *B*, and *C* showing excellent agreement with experimental values. In fact, the predicted values are accurate. In contrast, MP2 produces significantly poorer predictions, particularly for rotational constant *A*, which deviates substantially from experimental observations. This discrepancy arises from fundamental structural differences as shown in Figure 3: B3LYP (incorporating Grimme’s dispersion corrections D3) positions the N-H group pointing toward the π-electron density of the pyrrole ring but with the C=O group almost coplanar (5.9°) with the NH group, while MP2 has the amino group considerably out of coplanarity (13.9°). There is also a difference of 20° in the C-C-C-N dihedral angle. This subtle reorientation dramatically affects the rotational constants, highlighting the power of microwave spectroscopy for accurate structural determination. B2PLYP-D3BJ calculations confirm this trend, yielding intermediate values between B3LYP-D3BJ and MP2, with the optimized structure representing a geometric intermediate between the two extremes. Nuclear quadrupole coupling constants further validate this assessment, as they greatly depend on the chemical environment: as can be seen in Table 1, B3LYP-D3BJ provides accurate values closely matching experimental data, while MP2 predictions are poor for the amino group nitrogen, and B2PLYP-D3BJ values again fall intermediate between the two methods. These results conclusively demonstrate that the B3LYP-D3BJ-optimized structure best represents the true molecular geometry of 3-indoleacetamide.

We calculated the obtained parameters using selected basis sets (See Table S1). def2-tzvp basis set has minor consequences for B3LYP-D3BJ but improves the calculated values of B2PLYP-D3BJ being now accurate. This basis set also improves the rotational constants and quadrupole coupling constants for MP2 but still are far from being accurate. Finally, aug-cc-pvTZ basis set barely alters any of the predicted values for B3LYP-D3BJ and MP2 with respect to those calculated using def2-tzvp. For B2PLYP-D3BJ instead, while it does alter the rotational constants it has some minor effect on the nuclear quadrupole coupling constant to such extent that all calculated values are remarkably accurate.

Therefore, we conclude that B2PLYP-D3BJ/aug-cc-pvTZ offers the best prediction at the cost of being computationally expensive, while B3PLYP-D3BJ/6-311++G(d,p) offers excellent reproducibility particularly considering its low computational requirement.

Regarding nuclear quadrupole coupling constants, Table 2 shows a comparison of the nuclear quadrupole coupling constants with structurally related molecular systems to highlight how different environments affect these values. Using pyrrole as a reference, we observe that the nuclear quadrupole coupling constants of the nitrogen in the ring are drastically affected when a methyl group directly binds to the nitrogen (N-methylpyrrole). The same effect occurs with pyridine, which has extended aromaticity over a six-membered ring, where the nitrogen changes from having two bonds with carbons and one with hydrogen to only two carbon bonds that are more strongly bound due to the resonance effect. For indole, which consists of a six-membered benzene ring fused to a five-membered pyrrole ring, the values are not greatly affected due to the substitution being far from the nitrogen in the ring, although some noticeable differences remain. Finally, tryptamine has a 2-aminoethyl group attached to indole that interacts with the pyrrolic nitrogen. The nuclear coupling constants are similar but still slightly different from those of pyrrole and indole, with values falling between those of both systems. This demonstrates how small variations close to the nitrogen atom affect the quadrupole coupling constants. Comparing the quadrupole coupling constants of IAM with those of tryptamine confirms that the N_a_H•••N_r_ interaction is similar in both systems due to the very similar values observed for the pyrrolic nitrogen. On the other hand, the values for the amino nitrogen are drastically different due to the distinct chemical environments of the nitrogen in -CH_2_-NH_2_ for tryptamine and in -C=O-NH_2_ for IAM. In the next section, we provide further comparison of the latter through Non-Covalent Interaction (NCI) analysis.

### 2.4. Conformational Complexity Across the Tryptophan Family

**Figure 4:**
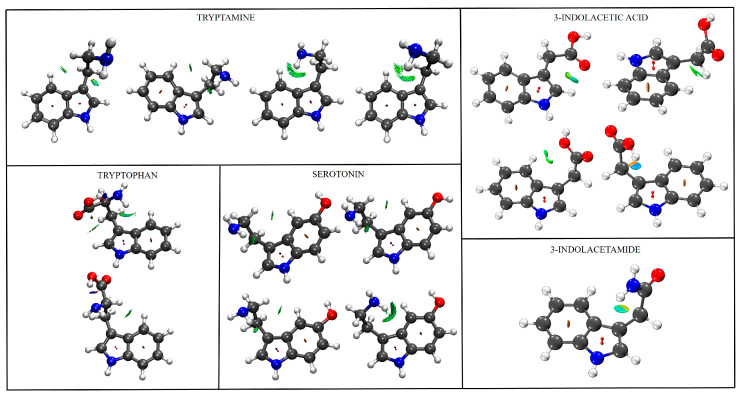
NCIPlot results of the detected conformers of tryptamine, tryptophan, serotonin, IAA, and IAM. Grey corresponds to carbons, blue to nitrogen, red to oxygen and white to hydrogen. Red surfaces correspond to repulsive forces, blue surfaces to moderate attractive forces, and green surfaces to weak attractive interactions. A contour value of 0.35 was used for the representation.

Table 3 shows the number of conformers and main intramolecular interactions for tryptamine, serotonin, tryptophan, IAA, and IAM, while Figure 4 shows a comparison of their structures including a NCIPlot [39,40]. Unlike the conformational diversity observed in related indole derivatives as explained in the introduction, where tryptamine and serotonin each adopt four distinct conformational states, tryptophan exhibits two stable conformers, and 3-indoleacetic acid presents four conformers with varying carboxyl orientations, 3-indoleacetamide demonstrates remarkable conformational rigidity, converging to a single stable structure. The conformational behavior of 3-indoleacetamide thus represents a dramatic departure from the flexibility observed in related tryptophan derivatives.

The unique single-conformer behavior of 3-indoleacetamide can be attributed to several key factors. The first factor is the amide constraint: unlike the flexible ethylamine chains in neurotransmitters or the rotatable carboxyl group in 3-indoleacetic acid, the acetamide group imposes geometric constraints that severely limit conformational freedom. The partial double-bond character of the C-N amide bond restricts rotation, while the quasi-planar geometry of the amide group defines specific spatial relationships. Another key factor is dual hydrogen bonding. The presence of amide double N-H functionality enables the formation of a stabilizing hydrogen bonding network. This dual interaction, one N-H with the carbonyl oxygen and another with the indole π-system, creates an energetically favorable “locked” conformation that is difficult to disrupt in sharp contrast with tryptamine. The third major factor is reduced degrees of freedom. The acetamide substituent eliminates many of the rotational degrees of freedom that contribute to conformational diversity in other tryptophan derivatives. The constrained geometry effectively “freezes” the molecule into its most stable arrangement.

Regarding the absence of other structural arrangements of IAM like those observed in tryptamine, serotonin, tryptamine or IAA, the quantum chemical calculations consistently converge to a single conformational structure, despite molecular mechanics identifying 12 potential conformers. This convergence can be attributed to several factors. The minor structural variants identified by molecular mechanics ultimately collapse to the global minimum when subjected to quantum mechanical optimization. Conformers featuring nearly in-plane arrangements, where the acetamide group adopts a colinear orientation with the indole system (although slightly out of plane), rapidly converge to the global minimum during geometry optimization. Notably, molecular mechanics identifies a conformer structurally similar to conformer IV of 3-indoleacetic acid; however, lacking a stabilization point, this structure also converges to the global minimum upon ab initio refinement. Finally, conformations where the acetamide group is positioned towards the π electronic density of the six-membered ring system (benzene) invariably collapse to the global minimum, indicating the dominant role of the N-H•••π (pyrrol) intramolecular interaction in determining the most stable geometry.

### 2.5. Structure-Function Relationships

While the molecules in Figure 1 and Figure 4 exhibit different acid-base properties in bulk aqueous solution (with tryptophan and 5-hydroxytryptophan existing as zwitterions, and tryptamine and serotonin as protonated amines), their behavior within enzyme active sites is governed by the local electrostatic environment of the binding pocket. In these different environment, the gas-phase results could provide essential information about the structure-property relationship within the pocket and benchmarks for computational modeling of enzyme-substrate complexes, where accurate conformational sampling is required prior to docking or molecular dynamics calculations in biological systems.

The conformational rigidity observed in 3-indoleacetamide may have profound implications for its biological role as an intermediate in auxin biosynthesis. The constrained structure may facilitate specific recognition by enzymes involved in the conversion to 3-indoleacetic acid. The rigid geometry could provide a “lock-and-key” fit with the active site, enhancing catalytic efficiency and specificity. Additionally, the reduced conformational flexibility may contribute to metabolic stability by limiting the number of reactive conformations accessible to degradative enzymes. In parallel, the conformational constraint limits the molecular orientations available for unwanted side reactions, potentially increasing the overall yield of the desired biosynthetic pathway. This could be particularly important for an intermediate that must be efficiently channeled through the biosynthetic pathway. Finally, the rigid structure may facilitate cellular transport and storage mechanisms, as the predictable geometry could enable specific interactions with transport proteins or storage complexes.

Although more results would be needed to provide a complete interpretation, the comparison with the two-step tryptophan-dependent serotonin and auxin biosynthesis pathways, as well as tryptamine, seem to indicate a correlation between their flexibility and biological role. Flexible molecules (serotonin, tryptamine) serve as neurotransmitters requiring adaptability for multiple receptor interactions or hormones with specific but varied biological roles (3-indoleacetic acid). Intermediate flexibility (tryptophan) on the other hand characterizes precursors. Finally rigid molecules (3-indoleacetamide) function as specialized biosynthetic intermediates requiring precise enzymatic recognition.

## 3. Materials and Methods

### 3.1. Experimental Methods

Solid rods of IAM (Cymit, m.p 150 °C) were prepared using commercial samples without further purification and pressing the compound’s fine powder mixed with a small amount of a commercial binder (Acryl 33). These rods were placed in the ablation nozzle, and a picosecond Nd: YAG laser (355 nm, 18 mJ per pulse, 20 ps pulse width) was used as a vaporization tool. The resulting products of the laser ablation were supersonically expanded utilizing a flow of neon gas at a backing pressure of 11 bar and then probed by Chirp Pulsed Fourier Transform Microwave (CP-FTMW) spectroscopy. Details of the experimental setup have been given elsewhere [26,27]. Chirped pulses of 4 µs directly generated by the 24 GS s^−1^ arbitrary waveform generator were amplified by a 200 W solid-state amplifier (SSA). The resulting pulses were then transmitted and detected by broadband microwave horn antennas in a high-vacuum chamber, where they interacted with the molecular supersonic expansion. At a repetition rate of 2 Hz, 96.000 free induction decays (4 FID emissions per gas pulse), each with a 10 µs length, were averaged and digitized using a 50 GS s^−1^ digital oscilloscope. The frequency-domain spectrum in the 7–13 GHz frequency range was obtained by taking a fast Fourier transform (FFT) following a Kaiser–Bessel window to improve the baseline resolution. The estimated accuracy of the frequency measurements is 30 kHz.

A laser ablation molecular beam Fourier transform microwave (LA-MB-FTMW) spectrometer [33,34] operating between 8 and 16 GHz was also used to resolve the hyperfine structure due to two ^14^N nuclei. A short microwave pulse with a duration of 0.3 µs and 1 dBm of power was applied to polarize the vaporized molecules. Fourier transform converted the detected free induction decay to the frequency domain. All transitions appear as Doppler doublets due to the coaxial configuration of the molecular beam and microwave radiation. The resonance frequency was determined as an arithmetic mean of the two Doppler components.

### 3.2. Computational Methods

The hindered rotations around the single bonds of IAM generate several conformational species. Therefore, the conformational space of IAM was first explored using fast molecular mechanics methods (MMFFs [41] forcefield) that implement two search algorithms “Large-scales Low-Mode” (which uses frequency modes to create new structures) and “Monte Carlo-based search algorithm”, as implemented in Macromodel [42].

Geometry optimizations of IAM were done using Gaussian suite programs [25]. The selected models for the primary investigation were an advanced DFT method based on a double-hybrid density functional (B2PLYP-D3BJ) with long-range dispersion corrections [43], a mixed method between Møller-Plesset (MP2) and DFT methods, with the Pople’s 6-311++G(d,p) basis set [44], as well as MP2 [45] and B3LYP-D3BJ [46,47,48] methodologies using the same basis set. def2-tzvp and aug-cc-pvTZ basis sets were also used for the three methodologies. Frequency calculations were also computed to ensure that the optimized geometries are true minima.

## 4. Conclusions

In this paper we provide a comprehensive conformational analysis of 3-indoleacetamide. The results show that there is a single stable conformer of IAM stabilized by an N–H•••O=C interaction between the amino and carbonyl groups of the side chain and a N–H•••π interaction between the amino NH_2_ group and the high π density sites of the pyrrolic ring.

This reveals unprecedented structural rigidity within the tryptophan-derived bioactive molecule family, as it represents a dramatic departure from the conformational flexibility characteristic of related neurotransmitters and plant hormones. The acetamide functional group imposes geometric constraints through restricted rotation due to partial double-bond character and the dual hydrogen bonding involving both N-H groups.

The conformational rigidity correlates with the molecule’s role as a biosynthetic intermediate rather than a final bioactive product.

This work opens several avenues for future investigation. We are currently working on the conformational analysis of other tryptophan-derived intermediates to test the generality of the rigidity-function relationship.

## Figures and Tables

**Table 1:** Experimental and calculated spectroscopic parameters for the detected conformer of IAM. The 6-311++G(d,p) basis set was employed. See Table S1 for other basis sets.

Parameters	Rot I		B3LYP-D3BJ	MP2	B2PLYP-D3BJ
	LA-CP-FTMW	LA-MB-FTMW			
*A* ^1^	1522.17866 (93) ^7^	1522.1819 (15) ^7^	1514	1445	1487
*B*	565.86458 (37)	565.86416 (38)	567	592	574
*C*	456.92186 (43)	456.92042 (55)	457	469	460
*μ_a_* ^2^	Observed	Observed	−4.2	−3.8	−4.0
*μ_b_*	Observed	Observed	−3.2	3.6	3.5
*μ_c_*	Observed	Observed	1.9	1.7	1.9
*χ_aa,r_* ^3^	1.446 (24)	1.4386 (66)	1.56	1.44	1.52
*χ_bb,r_*	1.608 (23)	1.5938 (64)	1.86	1.73	1.85
*χ_cc,r_*	−3.054 (23)	−3.0324 (64)	−3.43	−3.17	−3.36
*χ_aa,a_*	0.301 (35)	0.4469 (68)	0.57	−0.40	0.12
*χ_bb,a_*	−2.047 (28)	−2.0935 (64)	−2.40	−1.64	−2.15
*χ_cc,a_*	1.746 (28)	1.6466 (64)	1.84	2.04	2.03
Δ*_J_* ^4^	0.1114 (15)	0.1052 (28)	0.091	-	-
Δ*_JK_*	−0.5085 (54)	−0.524 (15)	−0.415	-	-
Δ*_K_*	1.653 (25)	1.792 (99)	1.407	-	-
*δ_J_*	0.03624 (65)	0.0358 (17)	0.029	-	-
σ ^5^	30	2.8	-	-	-
N ^6^	328	56	-	-	-

^1^ *A*, *B*, and *C* represent the rotational constants (in MHz); ^2^ *µ_a_*, *µ_b_* and *µ_c_* are the electric dipole moment components in Debyes (observed or not observed for experimental values). ^3^ *χ_aa_*, *χ_bb_*, and *χ_cc_*, are the diagonal elements of the ^14^N nuclear quadrupole coupling tensor (in MHz); N_r_ and N_a_ correspond to the ring and amine ^14^N nuclei, respectively. ^4^ Δ*_J_*, Δ*_K_*, Δ*_JK_*, and δ*_J_* are the quartic centrifugal distortion constants (in kHz). ^5^ RMS deviation of the fit (in kHz). ^6^ Number of measured transitions. ^7^ Standard error in parentheses expressed in units of the last digit.

**Table 2:** Comparison of the conformational behavior in some pyrrol and tryptophan derivatives.

Par.	IAM ^1^	Pyrrol ^2^	N-Methyl-Pyrrole ^3^	Pyridine ^4^	Indole ^5^	Tryptamine GPy-Out ^6^	Tryptamine GPh-Out ^6^
χ_aa,r_	1.4386 (66)	1.45 (2)	2.05 (5)	−4.88 (4)	1.7263 (43)	1.491 (17)	1.491 (87)
χ_bb,r_	1.5938 (64)	1.21 (2)	−1.69 (3)	1.43 (3)	1.6525 (50)	1.529 (14)	1.464 (37)
χ_cc,r_	−3.0324 (64)	−2.66 (2)	−0.37 (3)	3.45 (2)	−3.3788 (48)	−3.020 (14)	−2.955 (28)
χ_aa,a_	0.4469 (68)	-	-			−0.725 (19)	1.692 (33)
χ_bb,a_	−2.0935 (64)	-	-			−0.576 (19)	−0.331 (25)
χ_cc,a_	1.6466 (64)	-	-			1.301 (16)	−1.361 (25)

^1^ This work. ^2^ From reference [35]. ^3^ From reference [36], ^4^ From reference [37]. ^5^ From reference [38]. ^6^ From reference [18].

**Table 3:** Comparison of the conformational behavior in some tryptophan derivatives.

Molecule	Number of Conformers	Key Structural Features
Tryptamine ^1^	4	Ethylamine chain flexibility, N-H•π interaction (both rings)
Serotonin ^2^	4	Ethylamine chain flexibility, phenolic OH, N-H•π interaction (both rings)
IAA ^3^	4	Carboxyl group orientations, cis and trans, planar and out-of-plane, C-H•O and O-H•π interaction
Tryptophan ^4^	2	Trans-COOH, O-H•N and N-H•π interaction (both rings)
IAM ^5^	1	N-H•π interaction (pyrrol)

^1^ From Reference [18]. ^2^ From references [19,20]. ^3^ Article just submitted, ^4^ From references [21,22,23,24]. ^5^ This work.

## Data Availability

The original contributions presented in this study are included in the article. Further inquiries can be directed to the corresponding author.

## Supplementary Materials

The following supporting information can be downloaded at: https://www.mdpi.com/article/10.3390/molecules30214156/s1, Table S1: Experimental spectroscopic parameters of the detected conformer of IAM compared to the predicted values using different levels of theory and basis sets; Table S2: Measured frequencies and residuals for the rotational transitions of the detected conformer of IAM obtained with LA-CP-FTMW; Table S3: Measured frequencies and residuals for the rotational transitions of the detected conformer of IAM obtained with LA-MB-FTMW; Tables S4–S12: Cartesian coordinates in Angstroms (Å) of the detected conformer of IAM from the optimized structures at different levels of theory and basis set.
