# Supplementary material for: Conformational Analysis of 3-Indoleacetamide: Unveiling Structural Rigidity in the Tryptophan-Derived Bioactive Molecule Family
Source: Molecules. 2025 Oct 22;30(21):4156. doi: 10.3390/molecules30214156 (PMC12609830; doi:10.3390/molecules30214156)
Supplement: Supplementary file 1 [file molecules-30-04156-s001.zip › molecules-3925663-supplementary.pdf]

Supporting information for:

Conformational Analysis of 3-Indoleacetamide: Unveiling Structural Rigidity in the Tryptophan-Derived Bioactive Molecule Family

Sofía Municio, Sergio Mato, José Luis Alonso, Elena R. Alonso, Iker León*

AUTHOR ADDRESS

Sergio Mato - *Grupo de Espectrocopía Molecular (GEM), Edificio Quifima, Laboratorios de Espectroscopia y Bioespectroscopia, Unidad Asociada CSIC, Parque Científico UVa, Universidad de Valladolid, 47011, Valladolid, Spain*

Sofía Municio - *Grupo de Espectrocopía Molecular (GEM), Edificio Quifima, Laboratorios de Espectroscopia y Bioespectroscopia, Unidad Asociada CSIC, Parque Científico UVa, Universidad de Valladolid, 47011, Valladolid, Spain*

José L. Alonso - *Grupo de Espectrocopía Molecular (GEM), Edificio Quifima, Laboratorios de Espectroscopia y Bioespectroscopia, Unidad Asociada CSIC, Parque Científico UVa, Universidad de Valladolid, 47011, Valladolid, Spain*

Elena R. Alonso - *Grupo de Espectrocopía Molecular (GEM), Edificio Quifima, Laboratorios de Espectroscopia y Bioespectroscopia, Unidad Asociada CSIC, Parque Científico UVa, Universidad de Valladolid, 47011, Valladolid, Spain*

Corresponding Author

Iker León - *Grupo de Espectrocopía Molecular (GEM), Edificio Quifima, Laboratorios de Espectroscopia y Bioespectroscopia, Unidad Asociada CSIC, Parque Científico UVa, Universidad de Valladolid, 47011, Valladolid, Spain*
E-mail: Iker.leon@uva.es

Table S01. Experimental spectroscopic parameters of the detected conformer of IAM compared to the predicted values using different levels of theory and basis sets.

Parameters	Experimental	B3LYP-D3BJ	MP2	B2PLYP-D3BJ	B3LYP-D3BJ	MP2	B2PLYP-D3BJ	B3LYP-D3BJ	MP2	B2PLYP-D3BJ
	LA-MB-FTMW	6-311++G(d,p)			def2-tzvp			aug-cc-pvTZ		
A^1	1522.1819 (15)7	1514	1445	1487	1537	1500	1522	1531	1508	1522
B	565.86416 (38)	567	592	574	564	582	569	566	580	569
C	456.92042 (55)	457	469	460	457	466	459	457	465	459
$\mu_a{}^2$	Observed	-4.2	-3.8	-4.0	-4.1	-4.0	-4.1	-4.1	-4.1	-4.1
μ_b	Observed	-3.2	3.6	3.5	3.0	3.3	3.2	3.1	3.1	3.1
μ_c	Observed	1.9	1.7	1.9	2.0	1.9	2.0	1.9	1.8	1.9
$\chi_{aa,r}{}^3$	1.4386 (66)	1.56	1.44	1.52	1.47	1.27	1.43	1.45	1.25	1.41
$\chi_{bb,r}$	1.5938 (64)	1.86	1.73	1.85	1.75	1.54	1.71	1.71	1.41	1.62
$\chi_{cc,r}$	-3.0324 (64)	-3.43	-3.17	-3.36	-3.21	-2.81	-3.14	-3.16	-2.65	-3.03
$\chi_{aa,a}$	0.4469 (68)	0.57	-0.40	0.12	0.84	0.33	0.59	0.68	0.47	0.52
$\chi_{bb,a}$	-2.0935 (64)	-2.40	-1.64	-2.15	-2.38	-2.00	-2.27	-2.31	-1.80	-2.10
$\chi_{cc,a}$	1.6466 (64)	1.84	2.04	2.03	1.54	1.67	1.68	1.63	1.32	1.58
$\Delta_J{}^4$	0.1052 (28)	0.091	-	-	-	-	-	-	-	-
Δ_{JK}	-0.524 (15)	-0.415	-	-	-	-	-	-	-	-
Δ_K	1.792 (99)	1.407	-	-	-	-	-	-	-	-
δ_J	0.0358 (17)	0.029	-	-	-	-	-	-	-	-
σ^5	2.8	-	-	-	-	-	-	-	-	-
N^6	56	-	-	-	-	-	-	-	-	-

[1]*A*, *B*, and *C* represent the rotational constants (in MHz); [2]μ_a, μ_b and μ_c are the electric dipole moment components in Debyes (observed or not observed for experimental values). [3]χ_{aa}, χ_{bb}, and χ_{cc}, are the diagonal elements of the ^{14}N nuclear quadrupole coupling tensor (in MHz); N_r and N_a correspond to the ring and amine ^{14}N nuclei, respectively. [4]Δ_J, Δ_K, Δ_{JK}, and δ_J are the quartic centrifugal distortion constants (in kHz). [5]RMS deviation of the fit (in kHz). [6]Number of measured transitions. [7]Standard error in parentheses expressed in units of the last digit.

Table S02. Measured frequencies and residuals for the rotational transitions of the detected conformer of IAM obtained with LA-CP-FTMW.

J'	K'$_{-1}$	K'$_{+1}$	I'	F'	J''	K''$_{-1}$	K''$_{+1}$	I''	F'	ν_{obs}/MHz	$(\nu_{obs}-\nu_{calc})/MHz$
**7**	1	7	8	8	6	0	0	7	7	6988.33580	0.00433
**7**	1	7	8	9	6	0	6	7	8	6988.61670	0.02482
**7**	1	7	7	8	6	0	6	6	7	6988.90039	0.00311
**5**	1	4	5	4	4	0	4	4	3	7000.72770	-0.02143
**5**	1	4	5	6	4	0	4	4	5	7000.93312	0.01552
**5**	1	4	6	7	4	0	4	5	6	7001.99649	0.02399
**5**	1	4	4	5	4	0	4	3	4	7002.24930	0.01237
**5**	1	4	6	6	4	0	4	5	5	7002.75956	-0.03296
**5**	1	4	4	4	4	0	4	3	3	7003.04568	-0.00067
**4**	2	3	4	3	3	1	3	3	2	7447.73447	-0.00230
**4**	2	3	4	5	3	1	3	3	4	7447.95666	0.04745
**4**	2	3	4	4	3	1	3	3	3	7448.32086	0.02550
**4**	2	3	5	6	3	1	3	4	5	7448.79875	-0.03069
**4**	2	3	5	5	3	1	3	4	4	7449.39414	-0.01146
**4**	2	3	3	3	3	1	3	2	2	7449.71080	-0.01029
**4**	2	2	4	3	3	1	3	3	2	7575.52680	0.03196
**4**	2	2	4	5	3	1	3	3	4	7575.76270	0.02175
**4**	2	2	5	4	3	1	3	3	3	7576.13959	-0.00649
**4**	2	2	4	4	3	1	3	3	3	7576.42451	-0.02412
**4**	2	2	5	6	3	1	3	4	5	7576.98613	-0.02614
**4**	2	2	3	4	3	1	3	2	3	7577.52770	0.01271
**4**	2	2	5	5	3	1	3	4	4	7577.87759	-0.00579
**4**	2	2	3	3	3	1	3	2	2	7578.26534	-0.02176
**8**	1	7	8	9	7	0	7	7	8	11550.46561	-0.01775
**8**	1	7	9	10	7	0	7	8	9	11551.66381	-0.03081
**8**	1	7	9	9	7	0	7	8	8	11552.64405	-0.02217
**8**	1	7	7	7	7	0	7	6	6	11552.85540	0.01362
**6**	1	6	7	7	5	0	5	6	6	6177.60112	-0.02832
**6**	1	6	7	6	5	0	5	6	5	6178.03210	0.01931
**6**	1	6	6	5	5	0	5	5	4	6178.49019	0.05457
**6**	2	5	7	8	5	2	4	6	7	6097.24330	-0.01977
**6**	3	3	7	8	5	3	2	6	7	6197.63087	0.00392
**3**	2	2	3	4	2	1	2	2	3	6263.23128	-0.03774
**3**	2	2	3	3	2	1	2	2	2	6263.48609	0.06559
**3**	2	2	4	5	2	1	2	3	4	6264.19711	-0.01081
**3**	2	2	4	4	2	1	2	3	3	6264.74479	0.01308
**3**	2	2	2	2	2	1	2	1	1	6265.16710	0.00200
**3**	2	1	3	2	2	1	2	2	1	6306.39877	0.01250
**3**	2	1	3	4	2	1	2	2	3	6306.66814	0.02682
**3**	2	1	3	3	2	1	2	2	2	6306.90530	0.03438
**3**	2	1	4	5	2	1	2	3	4	6307.75182	-0.02384
**3**	2	1	4	4	2	1	2	3	3	6308.47511	0.01658

Table S02 (Continuation). Measured frequencies and residuals for the rotational transitions of the detected conformer of IAM obtained with CP-FTMW.

J'	K'$_{-1}$	K'$_{+1}$	I'	F'	J''	K''$_{-1}$	K''$_{+1}$	I''	F'	ν_{obs}/MHz	$(\nu_{obs}-\nu_{calc})/MHz$
**3**	2	1	2	2	2	1	2	1	1	6308.92175	-0.03670
**6**	1	5	7	8	5	1	4	6	7	6380.42531	0.04290
**7**	0	7	8	9	6	1	6	7	8	6485.56410	-0.06630
**7**	1	7	8	9	6	1	6	7	8	6687.65564	-0.03123
**7**	0	7	8	9	6	0	6	7	8	6786.57686	0.04146
**4**	2	3	3	3	3	1	2	2	2	6794.73540	-0.00519
**4**	2	3	5	5	3	1	2	4	4	6794.87060	-0.04007
**4**	2	3	5	6	3	1	2	4	5	6795.48670	-0.06938
**4**	2	3	5	4	3	1	2	4	3	6795.82343	-0.02171
**4**	2	3	4	5	3	1	2	3	4	6796.14696	0.00083
**4**	2	3	4	3	3	1	2	3	2	6796.37120	0.01317
**4**	2	2	5	5	3	1	2	4	4	6923.35414	-0.03431
**4**	2	2	5	6	3	1	2	4	5	6923.72372	-0.01519
**4**	2	2	4	5	3	1	2	3	4	6923.97957	0.00170
**4**	2	2	4	3	3	1	2	3	2	6924.18480	0.06871
**7**	2	6	8	9	6	2	5	7	8	7091.79909	0.01216
**7**	4	4	8	9	6	4	3	7	8	7202.30476	-0.01159
**7**	4	3	8	8	6	4	2	7	7	7204.53321	-0.04153
**7**	3	5	8	9	6	3	4	7	8	7207.35254	-0.04113
**7**	3	4	8	9	6	3	3	7	8	7260.24352	0.02177
**7**	1	6	8	9	6	1	5	7	8	7397.31229	-0.00960
**7**	2	5	8	9	6	2	4	7	8	7462.44756	0.05257
**8**	0	8	9	10	7	1	7	8	9	7487.86368	-0.02040
**5**	2	4	4	4	4	1	3	3	3	7599.11390	0.00111
**5**	2	4	6	6	4	1	3	5	5	7599.28413	0.02976
**5**	2	4	4	5	4	1	3	3	4	7599.79300	0.00972
**5**	2	4	6	5	4	1	3	5	4	7600.09005	-0.00289
**5**	2	4	5	6	4	1	3	4	5	7600.58593	0.04058
**5**	2	4	5	4	4	1	3	4	3	7600.73570	0.02918
**8**	1	8	9	10	7	1	7	8	9	7618.38992	-0.02866
**8**	0	8	9	10	7	0	7	8	9	7689.93189	-0.00867
**8**	1	8	9	10	7	0	7	8	9	7820.46587	-0.00920
**8**	1	8	8	9	7	0	7	7	8	7820.64850	-0.04615
**5**	2	3	6	7	4	1	3	5	6	7888.75995	0.06509
**8**	2	7	9	10	7	2	6	8	9	8076.97441	0.02005
**3**	3	1	4	5	2	2	0	3	4	8118.71338	-0.05131
**3**	3	0	4	5	2	2	0	3	4	8119.16283	-0.04672
**3**	3	1	4	5	2	2	1	3	4	8127.56229	0.00769
**3**	3	0	4	5	2	2	1	3	4	8128.00312	0.00366
**8**	3	6	9	10	7	3	5	8	9	8239.56044	-0.02822
**8**	4	5	9	10	7	4	4	8	9	8241.42882	-0.02895
**8**	4	4	9	10	7	4	3	8	9	8247.40669	-0.03226

Table S02 (Continuation). Measured frequencies and residuals for the rotational transitions of the detected conformer of IAM obtained with CP-FTMW.

J'	K'$_{-1}$	K'$_{+1}$	I'	F'	J''	K''$_{-1}$	K''$_{+1}$	I''	F'	ν_{obs}/MHz	$(\nu_{obs}-\nu_{calc})/MHz$
**8**	3	5	9	10	7	3	4	8	9	8340.54048	0.02729
**6**	2	5	7	7	5	1	4	6	6	8353.57039	-0.03804
**6**	2	5	7	8	5	1	4	6	7	8354.22038	-0.01967
**6**	2	5	6	7	5	1	4	5	6	8354.93171	0.02444
**8**	1	7	9	10	7	1	6	8	9	8388.70490	-0.03053
**6**	1	5	6	5	5	0	5	5	4	8427.91694	-0.03418
**6**	1	5	6	7	5	0	5	5	6	8428.15237	0.04221
**6**	1	5	7	8	5	0	5	6	7	8429.25280	0.00353
**6**	1	5	5	6	5	0	5	4	5	8429.50288	0.01294
**6**	1	5	7	7	5	0	5	6	6	8430.14080	-0.00919
**6**	1	5	5	5	5	0	5	4	4	8430.36450	-0.00861
**9**	0	9	10	11	8	1	8	9	10	8462.35028	-0.01203
**9**	1	9	10	11	8	1	8	9	10	8544.15371	-0.03871
**8**	2	6	9	10	7	2	5	8	9	8563.05726	-0.03259
**9**	0	9	10	11	8	0	8	9	10	8592.90414	0.00732
**9**	1	9	10	11	8	0	8	9	10	8674.68001	-0.04691
**5**	2	4	5	6	4	1	4	4	5	8685.34546	-0.02416
**5**	2	4	5	5	4	1	4	4	4	8685.88821	0.02671
**5**	2	4	6	7	4	1	4	5	6	8686.27968	0.00533
**5**	2	4	4	5	4	1	4	3	4	8686.60650	0.02576
**5**	2	4	6	6	4	1	4	5	5	8686.87155	-0.01475
**5**	2	4	4	4	4	1	4	3	3	8687.14190	0.01622
**6**	2	4	7	8	5	1	4	6	7	8901.88862	-0.01944
**5**	2	3	5	4	4	1	4	4	3	8973.40070	-0.01239
**5**	2	3	5	6	4	1	4	4	5	8973.63349	-0.01100
**5**	2	3	6	5	4	1	4	4	4	8974.20395	0.01840
**5**	2	3	5	5	4	1	4	4	4	8974.60037	0.00721
**5**	2	3	6	5	4	1	4	5	4	8974.87060	-0.00944
**5**	2	3	6	7	4	1	4	5	6	8975.10488	0.03499
**5**	2	3	4	5	4	1	4	3	4	8975.48623	0.01476
**5**	2	3	6	6	4	1	4	5	5	8976.10314	-0.01520
**5**	2	3	4	4	4	1	4	3	3	8976.43835	-0.02390
**10**	5	6	11	12	10	4	7	11	12	8998.00096	-0.02601
**9**	5	5	9	10	9	4	5	9	10	8999.31830	0.04258
**9**	5	4	10	11	9	4	5	10	11	8999.99071	-0.06261
**9**	5	5	10	11	9	4	6	10	11	9022.41466	-0.04135
**8**	5	3	9	10	8	4	4	9	10	9034.41154	-0.01852
**8**	5	4	9	10	8	4	5	9	10	9043.29864	0.05051
**9**	2	8	10	11	8	2	7	9	10	9052.16889	0.02398
**7**	5	2	8	9	7	4	3	8	9	9056.41704	-0.02615
**7**	5	3	8	8	7	4	4	8	8	9059.33671	-0.01447
**7**	2	6	8	8	6	1	5	7	7	9065.00060	-0.03908

Table S02 (Continuation). Measured frequencies and residuals for the rotational transitions of the detected conformer of IAM obtained with CP-FTMW.

J'	K'$_{-1}$	K'$_{+1}$	I'	F'	J''	K''$_{-1}$	K''$_{+1}$	I''	F'	ν_{obs}/MHz	$(\nu_{obs}-\nu_{calc})/MHz$
**7**	2	6	8	9	6	1	5	7	8	9065.62256	-0.02202
**7**	2	6	7	8	6	1	5	6	7	9066.34472	0.03952
**6**	5	1	6	7	6	4	2	6	7	9069.92004	0.00668
**6**	5	1	7	8	6	4	2	7	8	9070.23819	0.03512
**6**	5	2	6	7	6	4	3	6	7	9070.72235	-0.01508
**6**	5	2	7	8	6	4	3	7	8	9071.04022	0.01086
**5**	5	0	5	6	5	4	1	5	6	9078.11205	0.05095
**5**	5	0	6	7	5	4	1	6	7	9078.51120	0.03065
**4**	3	1	5	5	3	2	1	4	4	9126.07887	0.01888
**4**	3	1	4	5	3	2	2	3	4	9169.53384	0.01392
**4**	3	1	5	6	3	2	2	4	5	9169.74541	-0.02018
**10**	1	9	10	11	9	2	8	9	10	9227.82840	0.00005
**10**	1	9	11	12	9	2	8	10	11	9228.10495	0.02521
**10**	1	9	11	11	9	2	8	10	10	9228.40530	0.07841
**9**	8	1	10	11	8	8	0	9	10	9235.47404	0.01647
**9**	8	1	9	10	8	8	0	8	9	9235.73036	0.01843
**9**	7	2	10	11	8	7	1	9	10	9240.68279	0.02870
**9**	6	3	10	11	8	6	2	9	10	9248.82064	-0.04488
**9**	5	5	10	11	8	5	4	9	10	9262.65726	-0.02054
**9**	5	4	10	11	8	5	3	9	10	9263.15815	-0.01600
**9**	3	7	10	11	8	3	6	9	10	9267.94717	0.00545
**9**	4	5	10	11	8	4	4	9	10	9297.51872	-0.03215
**9**	1	8	10	11	8	1	7	9	10	9351.26779	-0.02507
**10**	0	10	11	12	9	1	9	10	11	9415.99876	-0.00610
**9**	3	6	10	11	8	3	5	9	10	9440.67688	0.05725
**10**	1	10	11	12	9	1	9	10	11	9466.09712	-0.02285
**10**	0	10	11	12	9	0	9	10	11	9497.84645	0.01149
**10**	1	10	11	12	9	0	9	10	11	9547.94962	-0.00045
**9**	2	7	10	9	8	2	6	9	8	9649.92560	0.01038
**8**	2	7	9	9	7	1	6	8	8	9744.69671	-0.01709
**8**	2	7	8	9	7	1	6	7	8	9745.90994	0.00462
**7**	1	6	7	8	6	0	6	6	7	9948.27845	-0.02007
**7**	1	6	8	9	6	0	6	7	8	9949.44090	-0.05368
**7**	1	6	6	7	6	0	6	5	6	9949.70940	-0.00578
**7**	1	6	8	8	6	0	6	7	7	9950.45264	0.00241
**7**	1	6	6	6	6	0	6	5	5	9950.65020	0.00075
**12**	2	10	13	14	11	3	9	12	13	9960.59002	0.07376
**12**	2	10	13	13	11	3	9	12	12	9960.95640	-0.01101
**6**	2	5	6	7	5	1	5	5	6	9975.15524	-0.03118
**6**	2	5	6	6	5	1	5	5	5	9975.75246	0.01596
**6**	2	5	7	8	5	1	5	6	7	9976.02344	-0.05734
**6**	2	5	5	6	5	1	5	4	5	9976.29348	-0.02451

Table S02 (Continuation). Measured frequencies and residuals for the rotational transitions of the detected conformer of IAM obtained with CP-FTMW.

J'	K'$_{-1}$	K'$_{+1}$	I'	F'	J''	K''$_{-1}$	K''$_{+1}$	I''	F'	ν_{obs}/MHz	$(\nu_{obs}-\nu_{calc})/MHz$
**6**	2	5	7	7	5	1	5	6	6	9976.68605	-0.02951
**6**	2	5	5	5	5	1	5	4	4	9976.89797	-0.00913
**7**	2	5	7	8	6	1	5	6	7	9983.72512	-0.03266
**7**	2	5	8	9	6	1	5	7	8	9983.97040	0.04976
**10**	2	9	11	12	9	2	8	10	11	10017.17840	0.01170
**5**	3	3	6	6	4	2	2	5	5	10093.68760	0.00989
**5**	3	3	6	7	4	2	2	5	6	10093.94710	0.03306
**5**	3	2	6	6	4	2	2	5	5	10106.01911	-0.00530
**5**	3	2	6	7	4	2	2	5	6	10106.22820	-0.00338
**5**	3	3	5	6	4	2	3	4	5	10221.84911	0.00486
**5**	3	3	6	7	4	2	3	5	6	10222.03940	-0.05747
**5**	3	2	5	6	4	2	3	4	5	10234.13584	0.00857
**5**	3	2	6	7	4	2	3	5	6	10234.47535	0.06094
**10**	9	1	11	12	9	9	0	10	11	10261.20910	0.01076
**10**	9	1	10	11	9	9	0	9	10	10261.42080	-0.01313
**10**	8	2	11	12	9	8	1	10	11	10265.97329	-0.02288
**10**	8	2	10	11	9	8	1	9	10	10266.16880	-0.01115
**10**	7	3	11	12	9	7	2	10	11	10273.12242	-0.03362
**10**	6	4	11	12	9	6	3	10	11	10284.45174	-0.02817
**10**	1	9	11	11	9	1	8	10	10	10285.26710	-0.09141
**10**	1	9	11	12	9	1	8	10	11	10285.48987	0.01647
**10**	3	8	11	12	9	3	7	10	11	10290.34114	0.01485
**10**	5	6	11	12	9	5	5	10	11	10303.16359	-0.04985
**10**	5	5	11	12	9	5	4	10	11	10304.54277	-0.04506
**10**	4	7	11	12	9	4	6	10	11	10327.66156	0.01908
**11**	0	11	12	13	10	1	10	11	12	10355.08613	-0.00551
**10**	4	6	11	12	9	4	5	10	11	10357.30678	-0.02507
**11**	1	11	12	13	10	1	10	11	12	10385.21004	-0.01567
**11**	0	11	12	13	10	0	10	11	12	10405.18176	-0.02498
**11**	1	10	11	12	10	2	9	10	11	10407.41055	-0.02034
**11**	1	10	12	13	10	2	9	11	12	10407.62659	0.02638
**9**	2	8	10	10	8	1	7	9	9	10408.22833	0.04784
**11**	1	11	12	13	10	0	10	11	12	10435.36255	0.02174
**6**	2	4	6	5	5	1	5	5	4	10521.94800	-0.00507
**6**	2	4	6	7	5	1	5	5	6	10522.16536	-0.00561
**6**	2	4	6	6	5	1	5	5	5	10523.30060	-0.01161
**6**	2	4	7	6	5	1	5	6	5	10523.56840	0.00934
**6**	2	4	7	8	5	1	5	6	7	10523.81881	0.07003
**6**	2	4	5	6	5	1	5	4	5	10524.09920	0.00810
**6**	2	4	7	7	5	1	5	6	6	10524.93581	-0.01963
**6**	2	4	5	5	5	1	5	4	4	10525.24330	-0.01763
**10**	3	7	11	12	9	3	6	10	11	10558.78684	0.05019

Table S02 (Continuation). Measured frequencies and residuals for the rotational transitions of the detected conformer of IAM obtained with CP-FTMW.

J'	K'$_{-1}$	K'$_{+1}$	I'	F'	J''	K''$_{-1}$	K''$_{+1}$	I''	F'	ν_{obs}/MHz	$(\nu_{obs}-\nu_{calc})/MHz$
**10**	2	8	11	12	9	2	7	10	11	10717.04924	0.02243
**17**	6	12	17	18	17	5	13	17	18	10777.05977	0.00100
**16**	6	11	17	18	16	5	12	17	18	10819.97035	0.00607
**14**	6	8	14	14	14	5	9	14	14	10857.33916	0.02094
**14**	6	9	15	16	14	5	10	15	16	10913.45255	0.00916
**13**	6	8	13	14	13	5	8	13	14	10927.36860	0.01960
**13**	6	7	14	15	13	5	8	14	15	10928.55895	-0.03307
**11**	2	10	11	11	10	2	9	10	10	10972.31453	-0.01252
**12**	6	6	13	14	12	5	7	13	14	10980.59160	0.00819
**6**	3	4	7	8	5	2	3	6	7	11012.40718	-0.01421
**11**	6	5	12	13	11	5	6	12	13	11018.60384	-0.01707
**11**	6	6	12	13	11	5	7	12	13	11023.87429	-0.04932
**10**	6	4	11	12	10	5	5	11	12	11046.37610	-0.01899
**10**	6	5	9	9	10	5	6	9	9	11048.31889	-0.04920
**6**	3	3	7	7	5	2	3	6	6	11048.63140	-0.00496
**6**	3	3	7	8	5	2	3	6	7	11048.82913	-0.07338
**6**	3	3	6	7	5	2	3	5	6	11049.08560	0.01339
**9**	6	3	10	11	9	5	4	10	11	11066.51087	0.00787
**9**	6	4	10	11	9	5	5	10	11	11067.20213	0.01375
**10**	2	9	11	11	9	1	8	10	10	11074.08596	-0.06236
**10**	2	9	10	11	9	1	8	9	10	11075.08009	0.04397
**8**	6	2	9	10	8	5	3	9	10	11080.79657	-0.01506
**8**	6	3	9	10	8	5	4	9	10	11081.02810	0.01835
**7**	6	1	7	8	7	5	2	7	8	11090.45750	0.01690
**7**	6	1	8	9	7	5	2	8	9	11090.69738	-0.01678
**6**	6	0	6	7	6	5	1	6	7	11096.93613	0.01165
**6**	6	0	7	8	6	5	1	7	8	11097.29231	-0.00397
**8**	2	6	8	9	7	1	6	7	8	11149.41485	0.03101
**8**	2	6	9	10	7	1	6	8	9	11149.72298	0.03438
**8**	2	6	9	9	7	1	6	8	8	11149.93750	0.03483
**4**	4	1	5	6	3	3	0	4	5	11167.64382	0.05591
**4**	4	0	5	6	3	3	1	4	5	11168.11690	0.06547
**11**	1	10	12	13	10	1	9	11	12	11196.65690	-0.03027
**12**	0	12	13	14	11	1	11	12	13	11284.49891	-0.00571
**11**	8	3	12	13	10	8	2	11	12	11297.88925	-0.01372
**6**	3	4	6	7	5	2	4	5	6	11300.95761	0.02143
**6**	3	4	7	8	5	2	4	6	7	11301.22522	0.00829
**12**	1	12	13	14	11	1	11	12	13	11302.35409	-0.00530
**11**	3	9	12	13	10	3	8	11	12	11304.81338	0.01218
**11**	7	4	12	13	10	7	3	11	12	11307.47665	-0.00024
**12**	0	12	13	14	11	0	11	12	13	11314.62224	-0.01646
**7**	2	6	7	8	6	1	6	6	7	11316.00383	-0.02353

Table S02 (Continuation). Measured frequencies and residuals for the rotational transitions of the detected conformer of IAM obtained with CP-FTMW.

J'	K'$_{-1}$	K'$_{+1}$	I'	F'	J''	K''$_{-1}$	K''$_{+1}$	I''	F'	ν_{obs}/MHz	$(\nu_{obs} - \nu_{calc})/\ MHz$
**7**	2	6	7	7	6	1	6	6	6	11316.65868	0.04721
**7**	2	6	8	9	6	1	6	7	8	11316.88230	-0.02996
**7**	2	6	6	7	6	1	6	5	6	11317.13053	0.02578
**7**	2	6	8	8	6	1	6	7	7	11317.55080	-0.00943
**7**	2	6	6	6	6	1	6	5	5	11317.71020	-0.00891
**11**	6	6	11	12	10	6	5	10	11	11322.55410	-0.06500
**12**	1	12	13	14	11	0	11	12	13	11332.46456	-0.02890
**11**	5	7	11	12	10	5	6	10	11	11347.07452	-0.01191
**11**	5	6	11	12	10	5	5	10	11	11350.45733	-0.01031
**11**	4	8	12	12	10	4	7	11	11	11372.84136	0.00796
**11**	4	7	12	13	10	4	6	11	12	11429.91211	-0.01837
**12**	1	11	13	14	11	2	10	12	13	11529.10923	0.02279
**11**	3	8	12	13	10	3	7	11	12	11688.00344	0.06489
**11**	2	9	12	13	10	2	8	11	12	11759.93026	0.00762
**11**	2	10	12	12	10	1	9	11	11	11761.01193	-0.02767
**11**	2	10	12	13	10	1	9	11	12	11761.35578	-0.03348
**11**	2	10	11	12	10	1	9	10	11	11761.78939	-0.00573
**12**	2	11	13	14	11	2	10	12	13	11918.28069	-0.00813
**12**	1	11	13	14	11	1	10	12	13	12093.79526	0.00674
**5**	4	2	6	7	4	3	1	5	6	12190.96211	0.02393
**5**	4	1	6	7	4	3	2	5	6	12194.20643	-0.00135
**13**	0	13	14	15	12	1	12	13	14	12207.71174	-0.00675
**13**	1	13	14	15	12	1	12	13	14	12218.16268	-0.00812
**13**	0	13	14	15	12	0	12	13	14	12225.56248	-0.01078
**13**	1	13	14	15	12	0	12	13	14	12236.01255	-0.01301
**12**	3	10	13	14	11	3	9	12	13	12309.77655	0.01475
**12**	8	4	13	13	11	8	3	12	12	12331.33864	-0.01932
**12**	7	6	13	13	11	7	5	12	12	12343.79768	-0.04058
**12**	6	7	13	13	11	6	6	12	12	12363.50060	0.04053
**12**	6	6	13	14	11	6	5	12	13	12363.75082	0.01851
**12**	5	8	12	13	11	5	7	11	12	12394.23303	0.02114
**12**	5	7	13	14	11	5	6	12	13	12401.80373	0.03392
**12**	4	9	12	13	11	4	8	11	12	12417.36852	0.02148
**12**	2	11	13	13	11	1	10	12	12	12482.74040	0.02331
**12**	2	11	11	10	11	1	10	10	11	12482.94351	-0.04814
**12**	2	11	12	13	11	1	10	11	12	12483.30298	-0.00879
**12**	4	8	13	14	11	4	7	12	13	12518.87349	0.04331
**8**	3	6	9	10	7	2	5	8	9	12640.84594	-0.02726
**12**	2	10	13	14	11	2	9	12	13	12774.57368	0.05983
**12**	3	9	13	14	11	3	8	12	13	12817.57274	0.01610
**13**	2	12	14	15	12	2	11	13	14	12856.18354	-0.04292
**13**	1	12	14	15	12	1	11	13	14	12985.72605	0.09401

Table S02 (Continuation). Measured frequencies and residuals for the rotational transitions of the detected conformer of IAM obtained with CP-FTMW.

J'	K'_{-1}	K'_{+1}	I'	F'	J''	K''_{-1}	K''_{+1}	I''	F'	ν_{obs}/MHz	$(\nu_{obs}-\nu_{calc})/MHz$
**14**	0	14	15	16	13	1	13	14	15	13127.05557	-0.01271
**14**	1	14	15	16	13	1	13	14	15	13133.11821	-0.00761
**14**	0	14	15	16	13	0	13	14	15	13137.49315	-0.02744
**14**	1	14	15	16	13	0	13	14	15	13143.56279	-0.01534
**6**	4	3	7	8	5	3	2	6	7	13208.40493	0.02812
**6**	4	2	7	8	5	3	3	6	7	13221.49930	-0.02910
**13**	3	11	14	15	12	3	10	13	14	13304.04784	0.00554
**13**	5	9	14	15	12	5	8	13	14	13444.43920	0.05250
**13**	4	10	13	14	12	4	9	12	13	13459.27097	0.02953
**14**	1	13	15	16	13	1	12	14	15	13878.32123	-0.00588
**6**	4	3	6	7	5	4	2	5	6	6166.50116	-0.01090
**6**	4	2	6	7	5	4	1	5	6	6167.16151	-0.01544
**7**	5	3	7	8	6	5	2	6	7	7190.88730	-0.03697
**8**	7	1	9	10	7	7	0	8	9	8209.81243	0.03440
**8**	7	1	8	9	7	7	0	7	8	8210.07529	0.02115
**8**	5	3	9	10	7	5	2	8	9	8225.40500	-0.02081
**6**	1	5	6	6	5	0	5	5	5	8429.02568	0.04247
**4**	3	2	5	5	3	2	1	4	4	9122.91613	-0.03108
**4**	3	2	4	5	3	2	2	3	4	9166.48221	0.05166
**8**	2	7	7	8	7	1	6	6	7	9745.18690	-0.00931
**8**	2	7	8	8	7	1	6	7	7	9745.34964	0.00576
**7**	4	4	8	9	7	3	5	8	9	7042.11692	-0.03674
**5**	4	2	5	6	5	3	3	5	6	7054.10251	0.03413
**5**	4	2	6	7	5	3	3	6	7	7054.42225	0.03740
**4**	4	0	5	6	4	3	1	5	6	7057.60141	-0.00277
**6**	3	4	7	8	5	3	3	6	7	6173.45236	-0.01099
**7**	6	1	7	8	6	6	0	6	7	7184.54435	0.06536
**11**	4	8	12	13	11	3	9	12	13	7164.87303	-0.00285
**11**	4	8	12	13	11	3	9	12	13	7164.87303	-0.00285
**14**	4	11	15	16	14	3	12	15	16	7637.03021	0.05332
**14**	3	12	15	16	14	2	13	15	16	7642.42634	-0.05856
**9**	1	8	10	11	8	2	7	9	10	7994.74159	-0.00967
**11**	5	6	12	13	11	4	7	12	13	8867.82858	0.01596
**10**	5	5	11	12	10	4	6	11	12	8947.29029	-0.01901

Table S03. Measured frequencies and residuals for the rotational transitions of the detected conformer of IAM obtained with LA-MB-FTMW.

J'	K'$_{-1}$	K'$_{+1}$	I'	F'	J''	K''$_{-1}$	K''$_{+1}$	I''	F'	ν_{obs}/MHz	$(\nu_{obs}-\nu_{calc})/\ MHz$
**5**	3	2	5	4	4	2	3	4	3	10234.12363	-0.00423
**5**	3	2	5	6	4	2	3	4	5	10234.14669	0.00545
**5**	3	2	5	5	4	2	3	4	4	10234.23105	0.00211
**5**	3	2	6	7	4	2	3	5	6	10234.43234	0.00465
**5**	3	2	4	4	4	2	3	3	3	10234.56796	-0.00309
**4**	3	1	4	5	3	2	2	3	4	9169.53408	0.00067
**4**	3	1	5	6	3	2	2	4	5	9169.77823	-0.00006
**4**	3	1	3	4	3	2	2	2	3	9169.84170	-0.00094
**9**	2	8	10	11	8	2	7	9	10	9052.14502	0.00111
**9**	2	8	9	10	8	2	7	8	9	9052.22315	0.00095
**7**	2	6	6	6	6	1	5	5	5	9064.94154	-0.00061
**7**	2	6	8	8	6	1	5	7	7	9065.03920	0.00039
**7**	2	6	8	9	6	1	5	7	8	9065.64408	0.00130
**7**	2	6	6	5	6	1	5	5	6	9065.55131	0.00336
**7**	2	6	8	7	6	1	5	7	6	9065.75151	0.00240
**7**	2	6	7	8	6	1	5	6	7	9066.29963	0.00068
**7**	2	6	7	6	6	1	5	6	5	9066.40217	0.00179
**5**	2	3	6	6	4	1	3	5	5	7888.48670	0.00141
**5**	2	3	5	5	4	1	3	4	4	7888.57459	0.00110
**5**	2	3	6	7	4	1	3	5	6	7888.70154	-0.00017
**5**	2	3	4	5	4	1	3	3	4	7888.67713	-0.00301
**5**	2	3	5	6	4	1	3	4	5	7888.82586	0.00003
**3**	3	1	3	3	2	2	0	2	2	8118.60476	0.00053
**3**	3	1	4	5	2	2	0	3	4	8118.77566	-0.00153
**3**	3	1	4	4	2	2	0	3	3	8118.69734	0.00420
**3**	3	0	4	4	2	2	0	3	3	8119.14168	0.00140
**3**	3	0	4	5	2	2	0	3	4	8119.22468	0.00263
**5**	3	3	6	6	4	2	2	5	5	10093.67703	0.00107
**5**	3	3	4	4	4	2	2	3	3	10093.64773	-0.00358
**5**	3	3	5	5	4	2	2	4	4	10093.75515	0.00130
**5**	3	3	4	5	4	2	2	3	4	10093.90652	-0.00184
**5**	3	3	5	4	4	2	2	4	3	10094.09027	0.00197
**5**	3	3	6	7	4	2	2	5	6	10093.92669	0.00176
**8**	2	7	8	9	7	1	6	7	8	9745.89455	-0.00397
**8**	2	7	8	7	7	1	6	7	6	9745.97756	-0.00103
**8**	2	7	9	10	7	1	6	8	9	9745.26944	-0.00512
**8**	2	7	8	8	7	1	6	7	7	9745.33779	0.00015
**8**	2	7	9	8	7	1	6	8	7	9745.35733	0.00314
**8**	2	7	9	9	7	1	6	8	8	9744.71188	-0.00152
**8**	2	7	7	7	7	1	6	6	6	9744.63863	0.00419
**9**	2	8	9	10	8	2	7	8	9	9052.22315	0.00095
**9**	2	8	8	8	8	2	7	7	7	9052.09619	-0.00459

Table S03(Continuation). Measured frequencies and residuals for the rotational transitions of the detected conformer of IAM obtained with MB-FTMW.

J'	K'$_{-1}$	K'$_{+1}$	I'	F'	J''	K''$_{-1}$	K''$_{+1}$	I''	F'	ν_{obs}/MHz	$(\nu_{obs}-\nu_{calc})/MHz$
**9**	2	8	10	11	8	2	7	9	10	9052.14502	0.00111
**8**	0	8	8	9	7	0	7	7	8	7690.05205	0.00017
**8**	0	8	9	10	7	0	7	8	9	7689.92998	-0.00126
**8**	0	8	7	8	7	0	7	6	7	7689.94463	0.00364
**8**	0	8	9	9	7	0	7	8	8	7689.83232	-0.00402
**8**	0	8	7	7	7	0	7	6	6	7689.84697	0.00197
**6**	2	5	5	5	5	1	4	4	4	8353.48621	-0.00386
**6**	2	5	5	6	5	1	4	4	5	8354.13074	-0.00242
**6**	2	5	7	8	5	1	4	6	7	8354.23961	-0.00033
**6**	2	5	7	7	5	1	4	6	6	8353.60937	0.00213
**4**	3	2	4	4	3	2	1	4	3	9122.87522	-0.00601
**4**	3	2	4	5	3	2	1	3	4	9123.06639	-0.00293
**4**	3	2	5	6	3	2	1	4	5	9123.10057	-0.00709
**4**	3	2	5	4	3	2	1	3	3	9123.22639	0.00359

Table S04. Cartesian coordinates in Angstroms (Å) of the detected conformer of IAM from the optimized structures at B3LYP-D3BJ/6-311++G(d,p).

I			
Atoms	x	y	z
C	2.955681000	0.176172000	0.327143000
C	1.698380000	0.735904000	0.093139000
C	0.584041000	-0.049059000	-0.305738000
C	0.746689000	-1.433445000	-0.467290000
C	1.994593000	-1.992324000	-0.236519000
C	3.087206000	-1.195161000	0.156530000
C	-0.539483000	0.844859000	-0.455373000
C	-0.079737000	2.100909000	-0.149980000
N	1.262766000	2.043402000	0.180460000
C	-1.926543000	0.463257000	-0.862316000
C	-2.742890000	-0.365714000	0.139234000
O	-3.768091000	-0.931011000	-0.200380000
N	-2.274667000	-0.386671000	1.417275000
H	3.799210000	0.786094000	0.629841000
H	-0.090020000	-2.054798000	-0.765568000
H	2.135874000	-3.059548000	-0.359022000
H	4.049793000	-1.661713000	0.329332000
H	-0.607212000	3.042111000	-0.147928000
H	1.833035000	2.833644000	0.428926000
H	-2.523147000	1.356719000	-1.068885000
H	-1.921401000	-0.117348000	-1.788826000
H	-2.752747000	-0.966281000	2.088898000
H	-1.362954000	-0.020304000	1.639150000

Table S05. Cartesian coordinates in Angstroms (Å) of the detected conformer of IAM from the optimized structures at B3LYP-D3BJ/def2-tzvp.

I			
Atoms	x	y	z
C	-2.952664000	0.190493000	0.328693000
C	-1.693758000	0.741025000	0.100415000
C	-0.587787000	-0.047603000	-0.306301000
C	-0.763512000	-1.425596000	-0.481886000
C	-2.012761000	-1.974539000	-0.256236000
C	-3.095907000	-1.174147000	0.144397000
C	0.540131000	0.837291000	-0.448086000
C	0.089495000	2.090316000	-0.129908000
N	-1.248935000	2.039799000	0.200683000
C	1.923345000	0.455016000	-0.855606000
C	2.751295000	-0.364770000	0.140552000
O	3.819655000	-0.847763000	-0.183871000
N	2.237318000	-0.485072000	1.390327000
H	-3.789603000	0.803904000	0.637787000
H	0.066091000	-2.051059000	-0.786093000
H	-2.163144000	-3.037734000	-0.389568000
H	-4.060913000	-1.633809000	0.312828000
H	0.623931000	3.026081000	-0.118878000
H	-1.812708000	2.830577000	0.453459000
H	1.914593000	-0.131636000	-1.777045000
H	2.517221000	1.345400000	-1.073062000
H	1.309506000	-0.162865000	1.602827000
H	2.731844000	-1.054763000	2.055453000

Table S06. Cartesian coordinates in Angstroms (Å) of the detected conformer of IAM from the optimized structures at B3LYP-D3BJ/ aug-cc-pvTZ.

I			
Atoms	x	y	z
C	-2.953415000	0.187899000	0.326331000
C	-1.694830000	0.739227000	0.100186000
C	-0.588390000	-0.047992000	-0.306093000
C	-0.762272000	-1.425654000	-0.483746000
C	-2.011430000	-1.975638000	-0.260553000
C	-3.095402000	-1.176613000	0.140209000
C	0.539236000	0.837361000	-0.445665000
C	0.088699000	2.089761000	-0.126922000
N	-1.250843000	2.039058000	0.201723000
C	1.921579000	0.454561000	-0.854841000
C	2.742713000	-0.377427000	0.135800000
O	3.792582000	-0.894898000	-0.200203000
N	2.248898000	-0.466661000	1.396942000
H	-3.790427000	0.799512000	0.634836000
H	0.066932000	-2.049610000	-0.788413000
H	-2.160857000	-3.037702000	-0.395168000
H	-4.059293000	-1.636720000	0.306833000
H	0.622067000	3.024977000	-0.114569000
H	-1.813728000	2.828501000	0.457662000
H	1.912758000	-0.122630000	-1.780831000
H	2.518440000	1.344400000	-1.061852000
H	1.332358000	-0.122354000	1.619413000
H	2.737357000	-1.042812000	2.059800000

Table S07. Cartesian coordinates in Angstroms (Å) of the detected conformer of IAM from the optimized structures at MP2/6-311++G(d,p).

I			
Atoms	x	y	z
C	-2.928767000	0.137893000	0.349034000
C	-1.685145000	0.725532000	0.070891000
C	-0.547433000	-0.044827000	-0.303877000
C	-0.677385000	-1.442357000	-0.450200000
C	-1.912006000	-2.026951000	-0.181805000
C	-3.029949000	-1.243409000	0.195031000
C	0.544991000	0.868426000	-0.498757000
C	0.052702000	2.130372000	-0.213691000
N	-1.277548000	2.041370000	0.146996000
C	1.941412000	0.496573000	-0.884172000
C	2.666731000	-0.379381000	0.140780000
O	3.501235000	-1.208628000	-0.192319000
N	2.372238000	-0.096729000	1.451038000
H	-3.788975000	0.735317000	0.641753000
H	0.176707000	-2.051530000	-0.738557000
H	-2.031581000	-3.101837000	-0.287745000
H	-3.979465000	-1.732383000	0.395951000
H	0.555910000	3.089050000	-0.241230000
H	-1.886756000	2.830704000	0.303465000
H	1.963035000	-0.056109000	-1.828844000
H	2.548926000	1.399803000	-1.021859000
H	1.497907000	0.365541000	1.654746000
H	2.720695000	-0.753242000	2.135237000

Table S08. Cartesian coordinates in Angstroms (Å) of the detected conformer of IAM from the optimized structures at MP2/def2-tzvp.

I			
Atoms	x	y	z
C	-2.936969000	0.151818000	0.324371000
C	-1.686398000	0.726986000	0.088030000
C	-0.560097000	-0.042484000	-0.305961000
C	-0.699300000	-1.431317000	-0.465122000
C	-1.939421000	-2.002988000	-0.231464000
C	-3.045514000	-1.220778000	0.158086000
C	0.541082000	0.857871000	-0.462361000
C	0.058514000	2.113473000	-0.159428000
N	-1.273856000	2.034019000	0.170066000
C	1.929314000	0.491794000	-0.857998000
C	2.701032000	-0.363848000	0.143336000
O	3.689126000	-0.998889000	-0.191792000
N	2.248117000	-0.315147000	1.423786000
H	-3.791310000	0.749678000	0.624169000
H	0.147317000	-2.041926000	-0.763166000
H	-2.066846000	-3.073047000	-0.350170000
H	-4.001118000	-1.702269000	0.332073000
H	0.571550000	3.064635000	-0.161460000
H	-1.859065000	2.817117000	0.412803000
H	1.943841000	-0.064359000	-1.798872000
H	2.530284000	1.392045000	-1.020340000
H	1.335677000	0.063683000	1.619903000
H	2.683385000	-0.929703000	2.093496000

Table S09. Cartesian coordinates in Angstroms (Å) of the detected conformer of IAM from the optimized structures at MP2/aug-cc-pvTZ.

I			
Atoms	x	y	z
C	-2.922196000	0.161480000	0.336726000
C	-1.678145000	0.738441000	0.070558000
C	-0.547062000	-0.034816000	-0.303303000
C	-0.674190000	-1.430639000	-0.406070000
C	-1.907713000	-2.004892000	-0.141973000
C	-3.019030000	-1.218144000	0.224605000
C	0.545972000	0.868497000	-0.498142000
C	0.055023000	2.131119000	-0.238097000
N	-1.276007000	2.052063000	0.100033000
C	1.936054000	0.500642000	-0.885389000
C	2.726819000	-0.299919000	0.145986000
O	3.757476000	-0.885754000	-0.156781000
N	2.237675000	-0.267579000	1.414727000
H	-3.778498000	0.761492000	0.618942000
H	0.174683000	-2.043846000	-0.685069000
H	-2.025440000	-3.078077000	-0.217490000
H	-3.967283000	-1.700323000	0.423159000
H	0.558855000	3.084317000	-0.275684000
H	-1.864288000	2.836841000	0.322069000
H	1.950278000	-0.097376000	-1.798086000
H	2.525349000	1.396584000	-1.092669000
H	1.304448000	0.066370000	1.583970000
H	2.684698000	-0.853283000	2.099845000

Table S10. Cartesian coordinates in Angstroms (Å) of the detected conformer of IAM from the optimized structures at B2PLYP-D3BJ/6-311++G(d,p).

I			
Atoms	x	y	z
C	-2.950921000	0.164092000	0.330640000
C	-1.695578000	0.729431000	0.084665000
C	-0.575350000	-0.052946000	-0.303181000
C	-0.726297000	-1.443500000	-0.444193000
C	-1.971121000	-2.008688000	-0.200848000
C	-3.071137000	-1.212602000	0.182255000
C	0.539303000	0.846439000	-0.472194000
C	0.068082000	2.106233000	-0.186273000
N	-1.272128000	2.041871000	0.149219000
C	1.928348000	0.466259000	-0.876035000
C	2.719756000	-0.368947000	0.136689000
O	3.660093000	-1.066327000	-0.210470000
N	2.346915000	-0.222018000	1.442177000
H	-3.799111000	0.772005000	0.624611000
H	0.114498000	-2.063015000	-0.736176000
H	-2.104509000	-3.078737000	-0.306388000
H	-4.029687000	-1.684001000	0.364338000
H	0.586368000	3.052898000	-0.202811000
H	-1.850107000	2.831733000	0.381424000
H	1.928683000	-0.111086000	-1.803579000
H	2.525272000	1.363232000	-1.067939000
H	1.453416000	0.186404000	1.664536000
H	2.780410000	-0.832408000	2.116817000

Table S11. Cartesian coordinates in Angstroms (Å) of the detected conformer of IAM from the optimized structures at B2PLYP-D3BJ/def2-tzvp.

I			
Atoms	x	y	z
C	-2.949732000	0.177728000	0.326587000
C	-1.692805000	0.734552000	0.093750000
C	-0.580672000	-0.049179000	-0.303778000
C	-0.744525000	-1.431546000	-0.468179000
C	-1.991125000	-1.987185000	-0.238350000
C	-3.081698000	-1.190467000	0.154372000
C	0.539178000	0.840980000	-0.454594000
C	0.077383000	2.095447000	-0.148589000
N	-1.259568000	2.037305000	0.182242000
C	1.924482000	0.463975000	-0.858964000
C	2.736201000	-0.363601000	0.139795000
O	3.765822000	-0.922664000	-0.197322000
N	2.263750000	-0.391805000	1.412382000
H	-3.791718000	0.787150000	0.628829000
H	0.089534000	-2.053539000	-0.767389000
H	-2.133880000	-3.052199000	-0.362254000
H	-4.043039000	-1.655786000	0.325924000
H	0.603504000	3.036052000	-0.147181000
H	-1.830531000	2.826413000	0.424921000
H	1.923405000	-0.113885000	-1.784853000
H	2.518245000	1.358378000	-1.059835000
H	1.348206000	-0.038591000	1.627568000
H	2.740298000	-0.975404000	2.078184000

Table S12. Cartesian coordinates in Angstroms (Å) of the detected conformer of IAM from the optimized structures at B2PLYP-D3BJ/aug-cc-pvTZ.

I			
Atoms	x	y	z
C	-2.942886000	0.175113000	0.332478000
C	-1.691366000	0.736081000	0.081703000
C	-0.573827000	-0.048030000	-0.298798000
C	-0.725771000	-1.435691000	-0.425853000
C	-1.967110000	-1.995932000	-0.178164000
C	-3.063558000	-1.198477000	0.196799000
C	0.538508000	0.846969000	-0.475023000
C	0.067988000	2.105343000	-0.200344000
N	-1.268678000	2.045228000	0.134135000
C	1.925302000	0.470370000	-0.874403000
C	2.746234000	-0.324152000	0.142643000
O	3.781075000	-0.884241000	-0.182335000
N	2.277523000	-0.325128000	1.417728000
H	-3.788336000	0.784264000	0.620685000
H	0.111682000	-2.057712000	-0.710909000
H	-2.100860000	-3.063839000	-0.273233000
H	-4.019314000	-1.666777000	0.383032000
H	0.585650000	3.049000000	-0.224146000
H	-1.843714000	2.834618000	0.362090000
H	1.925157000	-0.131488000	-1.783396000
H	2.511034000	1.362430000	-1.100714000
H	1.360834000	0.025805000	1.626992000
H	2.759629000	-0.887433000	2.096317000
